# Simultaneous optogenetic manipulation and calcium imaging in freely moving *C. elegans*

**DOI:** 10.3389/fncir.2014.00028

**Published:** 2014-03-24

**Authors:** Frederick B. Shipley, Christopher M. Clark, Mark J. Alkema, Andrew M. Leifer

**Affiliations:** ^1^Lewis Sigler Institute for Integrative Genomics, Princeton UniversityPrinceton, NJ, USA; ^2^Department of Neurobiology, University of Massachusetts Medical SchoolWorcester, MA, USA

**Keywords:** optogenetics, calcium imaging, sensorimotor transformation, mechanosensation, behavior

## Abstract

Understanding how an organism's nervous system transforms sensory input into behavioral outputs requires recording and manipulating its neural activity during unrestrained behavior. Here we present an instrument to simultaneously monitor and manipulate neural activity while observing behavior in a freely moving animal, the nematode *Caenorhabditis elegans*. Neural activity is recorded optically from cells expressing a calcium indicator, GCaMP3. Neural activity is manipulated optically by illuminating targeted neurons expressing the optogenetic protein Channelrhodopsin. Real-time computer vision software tracks the animal's behavior and identifies the location of targeted neurons in the nematode as it crawls. Patterned illumination from a DMD is used to selectively illuminate subsets of neurons for either calcium imaging or optogenetic stimulation. Real-time computer vision software constantly updates the illumination pattern in response to the worm's movement and thereby allows for independent optical recording or activation of different neurons in the worm as it moves freely. We use the instrument to directly observe the relationship between sensory neuron activation, interneuron dynamics and locomotion in the worm's mechanosensory circuit. We record and compare calcium transients in the backward locomotion command interneurons AVA, in response to optical activation of the anterior mechanosensory neurons ALM, AVM or both.

## 1. Introduction

Understanding the neural basis of behavior is a fundamental goal of neuroscience. In many cases, however, the normal operation of neural circuits can be studied only in freely behaving animals.

Previous neurophysiology experiments in behaving animals employed one of two approaches: Either probes, such as electrodes or optical fibers, were surgically implanted into an animal and recorded via tether or wireless backpack (Wilson and McNaughton, 1993; Lee et al., 2006; Szuts et al., 2011); or animals were head-fixed so that they were sufficiently immobile for electrical recording or microscopy Harvey et al. (2009); Seelig et al. (2010).

The nematode *Caenorhabditis elegans*, due to its small size and optical transparency allows for a third approach, whereby an external and non-invasive tracking microscope keeps pace with the worm's motion to image the worm brain while allowing the animal to roam freely without restraint. Moreover, the worm's small nervous system of 302 neurons, its genetic tractability and its known connectome make it well suited to an investigation of the neural basis of behavior.

Early recordings of neural activity from freely moving *C. elegans* used the genetically encoded calcium indicator Cameleon and required the experimenter to manually adjust a microscope stage to keep the worm centered under a microscope objective (Clark et al., 2007). Subsequently, automated tracking systems were developed that used computer vision (Ben Arous et al., 2010) or analog methods (Faumont et al., 2011) to track the worm's body motion automatically by adjusting a motorized stage. For many of these systems, intracellular calcium transients can be recorded while also observing the worm's behavior. Similar systems have been employed to measure neural activity in zebrafish larvae, another small optically transparent organism (Naumann et al., 2010; Muto et al., 2013). These systems have provided a valuable means to correlate activity with behavior and in worms they have elucidated neural coding of temperature during thermotaxis (Clark et al., 2007) and provided insights into neural dynamics correlated with transitions between forward and backward locomotion (Kawano et al., 2011; Piggott et al., 2011).

Optogenetics allows for optically stimulating or inhibiting neurons that express light activated proteins, like Channelrhodopsin. *C. elegans* was the first organism to have its behavior manipulated optogenetically (Nagel et al., 2005). Early experiments relied on genetic specificity for targeting their stimulus. For example, optogenetics was first used to study the mechanosensory circuit in *C. elegans*, but only through simultaneous stimulation of all touch receptor neurons, because promoters specific to each neuron are unavailable. Patterned illumination overcomes this limitation by combining genetic specificity with optical targeting. By delivering light to only targeted cells or tissues, neurons can be illuminated individually provided that there is a sufficiently sparse expression pattern. Previously we developed an optogenetic illumination system that allows perturbations of neural activity with high spatial and temporal resolution in an unrestrained worm, enabling us to control locomotion and behavior in real time (CoLBeRT) in *C. elegans* (Leifer et al., 2011). The CoLBeRT system and others like it (Stirman et al., 2011) have been instrumental in defining neural coding of several behaviors in *C. elegans* including chemotaxis (Kocabas et al., 2012), nociception (Husson et al., 2012) and the escape response (Donnelly et al., 2013).

We sought to combine these capabilities and thus simultaneously manipulate and monitor neural activity while also observing behavior.

Here we present an instrument that can perturb a neural circuit and immediately observe its effects both on behavior and the activity of other neurons in the circuit. This instrument integrates the functionality of the CoLBeRT tool with that of a previously developed calcium imaging instrument (Leifer, 2012) to enable simultaneous manipulation and monitoring of neural activity in sparsely labeled neural circuits. We used this combined system to investigate the sensorimotor transformation between mechanosensory stimulus, interneuron activity and behavior in the *C. elegans* escape response circuit. Using this tool we are able to stimulate individual mechanosensory neurons and directly observe the effect on a downstream command interneuron and on the worm's behavior, an experiment that would not be possible with previous techniques.

The integration of optogenetics, calcium imaging and behavioral analysis allows us to dissect neural circuit dynamics and correlate neural activity with behavior. To our knowledge, this is the first instrument, in any organism, to allow simultaneous non-invasive manipulation and monitoring of neural activity in an unrestrained freely moving animal.

## 2. Results

### 2.1. Experimental setup

**Figure 1:**
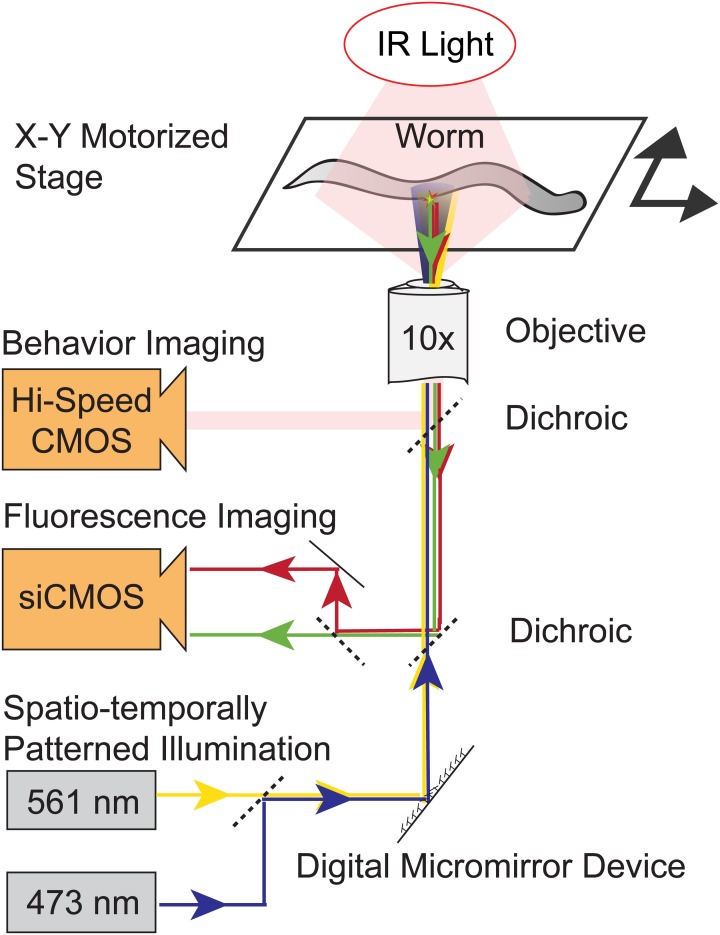
**Schematic of the illumination and imaging systems.** A worm moves freely on a motorized stage under infrared illumination (IR). A digital micromirror device (DMD) reflects blue and yellow laser light onto only targeted neurons. Three separate imaging paths simultaneously image the worm's behavior and record simultaneous red and green fluorescence images. Real-time computer vision software monitors the worm's posture and location and controls the DMD, lasers and stage. The DMD's illumination pattern is continuously updated to illuminate only targeted neurons.

*C. elegans* crawls freely on agarose on a motorized x-y translation stage under dark-field near-infrared (NIR) illumination, Figure 1. A high-speed behavior camera records the worm's position and orientation and real-time computer vision software extracts the outline of the worm and uses it to identify the worm's head, tail and centerline and the expected location of targeted neurons. At the heart of the instrument lies a DMD that generates patterned illumination targeted to individual neurons. Every ~13 ms (75 frames per second) the DMD adjust its mirrors to reflect blue and yellow laser-light only onto targeted neurons. For calcium imaging, the DMD reflects blue and yellow light onto neurons co-expressing the calcium indicator GCaMP3 and a calcium-insensitive fluorescent reference, mCherry. A dedicated imaging path simultaneously records two images of green and red fluorescence from GCaMP3 and mCherry, respectively. To stimulate neurons, the DMD adjusts its mirrors to also reflect laser light onto other cells expressing ChR2.

To accurately illuminate targeted neurons and not nearby neighbors, it is important to rapidly update the DMD's illumination pattern in response to the worm's change of position. The latency between a change in worm position and a corresponding change in illumination output is an important metric of the system's capabilities and represents the cumulative delay from a train of processes, including the time required to: expose one frame of the behavior camera, read out a frame from the camera and load it into software, perform computer vision analysis to extract the worm's position and orientation, send instructions to the DMD and finally adjust the DMD's mirrors. To measure this cumulative latency, we used an additional high speed camera to simultaneously optically record the image that is the input to our instrument and the position of the DMD's mirrors which is the system's output. Instead of an actual worm we used a computer monitor to display a stationary image of a worm that we could then manipulate precisely. In response to a step-wise translation of the image of the worm on our computer monitor (the input), the system updated the DMD mirrors (the output) in 28.6 ± 3 ms (mean ± standard deviation, *n* = 3 trials, measured with a camera recording 198 frames per second).

The low-latency observed in the system is due in part to the high speed open-source MindControl computer-vision software package, written in C, which rapidly performs computer vision analysis. We have updated this software with new features for timed stimuli, improved tracking and better stability. Source code is available online at http://github.com/leiferlab/mindcontrol.

### 2.2. Targeted illumination and fluorescent imaging in moving worms

To first validate the instrument, we sought to independently illuminate neurons in a moving worm. We generated a transgenic worm expressing GFP in the six soft-touch mechanosensory neurons, the ALM pair (ALML, ALMR), AVM, PVM, and the PLM pair (PLML, PLMR) and in the command interneuron pair AVA (AVAL and AVAR). Additionally, the promotor for AVA also expresses in nearby pharyngeal neurons (I1, I4, M4, NSM). By adjusting the illumination pattern, we illuminated the cell bodies of either the ALM neuron pair, the neuron AVM, or the AVA neuron pair along with nearby pharyngeal neurons, and we also illuminated different combinations thereof. We recorded fluorescent images as the worm crawled freely. Although these three targets were as close as 50 μm from one another, we observed no instances of errant illumination, see Supplementary Movie 1.

### 2.3. Optogenetic stimulation of single touch receptor cell types and simultaneous Ca^2+^ imaging

Calcium levels of AVA have been shown to increase during spontaneous bouts of backward locomotion (Chronis et al., 2007; Guo et al., 2009; Ben Arous et al., 2010; Kawano et al., 2011) and during mechanosensory evoked reversals (Leifer, 2012). Three mechanosensory neurons in the worm's anterior, the ALM pair and the neuron AVM, mediate the reversal response to soft touch (Chalfie and Sulston, 1981). Previously we showed that a brief optogenetical stimulation to either the neuron AVM or the neuron pair ALM was sufficient to induce a reversal (Leifer et al., 2011). During mechanical stimulation, however, all three of the anterior touch neurons are presumed to be activated.

**Figure 2:**
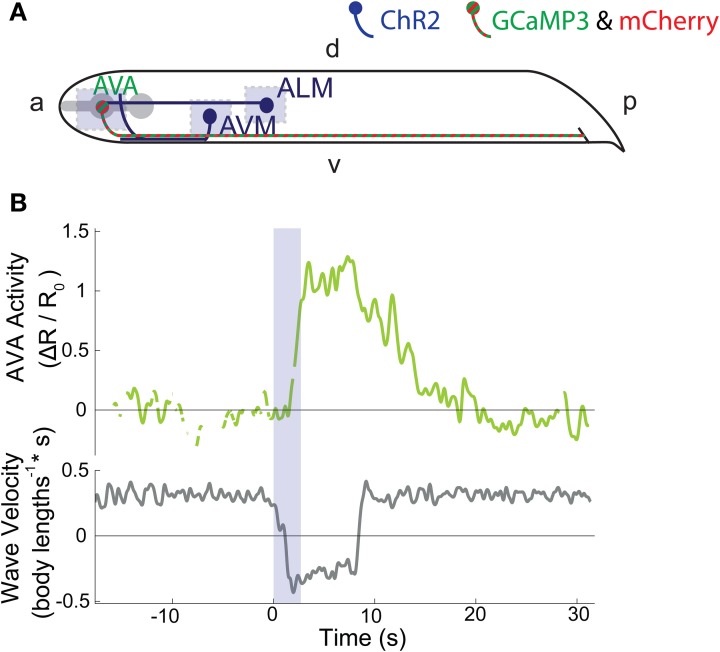
**(A)** ChR2 is expressed in six soft-touch mechanosensory neurons, including ALM and AVM (dark blue). Calcium indicator GCaMP3 and fluorescent reference mCherry are expressed in command interneuron AVA (striped green and red). Light blue shaded regions indicate areas of illumination. “a” is anterior, “p” is posterior, “d” is dorsal, and “v” is ventral. **(B)** Intracellular calcium dynamics of AVA (green line) are measured before during and after optogenetic stimulation of the ALM touch neuron (2.7 s stimulation, blue shaded region). The velocity of the worms body bending waves is shown in gray.

We asked whether the interneuron AVA exhibits elevated calcium transients during reversals caused by the stimulation of only a single mechanosensory neuron type. Moreover, we sought to investigate how the interneuron AVA integrates signals from ALM and AVM and how those signals drive the animal's behavioral response. We generated a transgenic line expressing ChR2 in the mechanosensory neurons and GCaMP3 and mCherry in the interneuron pair AVA. We stimulated either ALM, AVM or both neuron types Figure 2A by illuminating them for 2.7 s (*n* = 14, 12, 13 trials, respectively) in worms that were undergoing forward locomotion. We recorded calcium transients in AVA for at least 15 s before, during and after stimulus.

In these experiments the DMD adjusts a subset of its mirrors such that blue and yellow light continuously illuminates GCaMP3 and mCherry in AVA. Only during stimulation does the DMD transiently turn on other mirrors to illuminate neurons ALM and AVM which express ChR2. In this manner, AVA illumination is controlled independently of ALM and AVM illumination.

For all worms that reversed in response to stimulus, we observed elevated calcium levels in AVA immediately following stimulation of either ALM, AVM or both. Figure 2B and Supplementary Movie 2 show an example trace of AVA calcium activity in response to ALM stimulation. All traces for the combined ALM and AVM stimulation are shown in Supplementary Figure 1.

**Figure 3:**
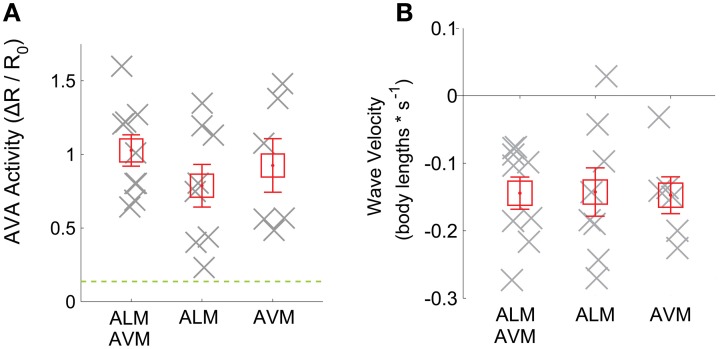
**(A)** Mean of AVA activity and **(B)** worm velocity during a time window are shown (gray crosses) for trials when the worm reversed in response to optogenetic illumination of ALM (*n* = 8 trials), AVM (*n* = 6 trials) or both (*n* = 9). Mean across trials is shown (red squares). Error bars represent standard error of the mean. The dashed green line shows the mean plus standard deviation of the apparent calcium signal observed during reversals in worms expressing calcium-insensitive GFP instead of GCaMP3 (*n* = 5 trials).

To reject the null hypothesis that our apparent calcium signals arise from instrument noise or motion artifact, we compared the calcium transients observed from GCaMP3 with apparent calcium transients observed from control worms that instead expressed calcium-insensitive GFP (*n* = 5 trials), see Figure 3A, (green dashed line) and Supplementary Figure 2. During reversals, AVA's activity as measured with GCaMP3, was more than one standard deviation above the mean greater than the apparent activity observed in calcium-insensitive GFP worms. This suggests that the observed signals reflect underlying calcium dynamics rather than motion artifacts or noise.

**Figure 4:**
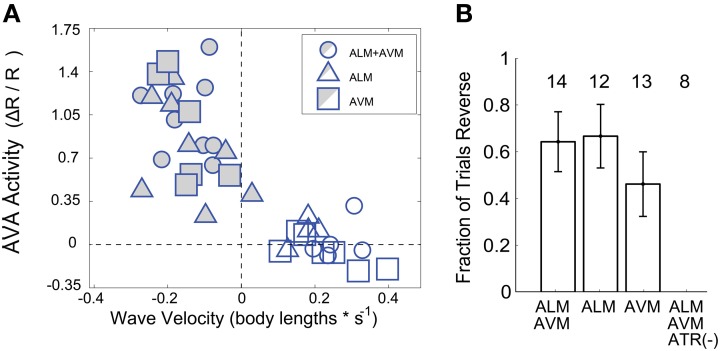
**(A)** For each trial, the amplitude of AVA activity is plotted against the within-trial mean velocity during a trial-specific time window, (*n* = 39 trials). Both reversers (shaded points) and non-reversers (open points) are included. **(B)** The fraction, *f*, of worms that respond by reversing (reversers) are shown for each stimuli. Number, *n*, indicates total trials. Control worms grown without the ChR2 co-factor all-trans retinal (ATR (−)) are also shown. Error bars are standard error, $\sigma_{f}=\sqrt{f(1-f)/n}$, where *f* is the fraction of worms that reverse out of a total, *n*.

When averaging across trials, neither AVA's response amplitude Figure 3A, nor the worm's reversal velocity Figure 3B were observed to vary significantly whether ALM or AVM or both were stimulated. The fraction of worms that responded to stimulus by reversing also did not vary significantly with stimulus but did depend, as expected, on the presence of the ChR2 cofactor all-trans retinal Figure 4B. Interestingly, however, the amplitude of AVA's activity inversely correlates with the worm's velocity immediately post stimulus (Pearsons correlation coefficient *R* = −0.83), irrespective of stimulus Figure 4A. Thus our data indicate that AVA's activity directly correlates with the worm's velocity and not with whether ALM, AVM or both are stimulated.

## 3. Materials and methods

### 3.1. Strains

Strain AML1 used in our experiment was made by crossing QW309 *(zfIs18)* [P*mec-4::ChR2::YFP*] with QW625 *(zfIs42)* [P*rig-d::GCaMP3::SL2::mCherry*]. Control experiments were performed with QW1075 *(zfEx416)* [P*rig-3::GFP:SL2::mCherry*], *(zdIs5)* [P*mec-4::GFP*].

### 3.2. Microscopy

The optogenetic stimulation and calcium imaging system was built around a modified Nikon Eclipse Ti-U inverted microscope that contained two stacked filter cube turrets. All imaging was conducted using a 10x, numerical aperture (NA) 0.45, Plano Apo objective. Worms were imaged on 6 cm NGM agarose plates on a Ludl BioPrecision2 XY motorized stage controlled by a MAC 6000 stage-controller. Custom real-time computer vision software kept the worm centered in the field of view via an automated feedback loop.

### 3.3. Behavioral imaging

Worm behavior was imaged on a CMOS camera (Basler aca2000-300 km) under dark-field illumination in the near-infrared (NIR). Dark-field illumination was chosen to provide high contrast between the worm and the agarose plate. NIR illumination was chosen to avoid cross talk with simultaneous fluorescence imaging and also to minimized inadvertent ChR2 activation. An NIR filter (Semrock #FF01-795/150), was mounted in the illumination path of a Nikon halogen lamp. Dark-field illumination was obtained by using a Ph3 phase ring to create an annular pattern of illumination. A custom filter cube (Semrock #FF670-SDi01) in the microscopes upper filter cube turret reflected the behavior imaging path out of the microscope through what would normally be its epifluorescence illumination pathway. From there, a telescope composed of two achromat doublet plano-convex lenses was used to form an image on the camera. A long-pass filter (Omega Optical #3RD710LP) prevented stray light from entering the camera. The Basler camera transferred images to a desktop computer via a PCI Express x16 10-tap Full Camera Link framegrabber (BitFlow Karbon). Images were recorded via the MindControl software discussed below.

### 3.4. Spatio-temporally patterned laser illumination

To stimulate ChR2 and to illuminate GCaMP3, we used a 473-nm diode-pumped solid state laser (DPSS) (CNI Laser MBL-III-473, 150-mW maximum power, OptoEngine). Similarly, to illuminate mCherry we used a 561-nm DPSS laser (Sapphire 561-150 CW CDRH, 150-mW maximum power, Coherent). Laser illumination entered the microscope from the bottom filter cube turret. The beams from the 473-nm and 561-nm lasers were first aligned to a common beam path by a series of mirrors and a dichroic mirror (Semrock #FF518-Di01). The combined beam path was then expanded using a telescope of two plano-convex achromat doublet lenses and reflected by a 2-inch diameter mirror onto a 1024 × 768 element DMD (Texas Instruments DLP, Discovery 4100 BD VIS 0.7-inch XGA, Digital Light Innovations). The patterned light from the DMD was imaged onto the sample via an achromat doublet that served as a tube lens, and a custom filter cube (Chroma #59022bs dichroic, Semrock #FF01-523/610 emission filter) in the microscopes lower filter cube turret. Light intensity measured at the sample was 2 mW * mm^−2^ of 473-nm light and 1 mW * mm^−2^ of 561-nm light.

The micromirrors of the system are imaged to the sample using a 10x objective such that each independent mirror element corresponds to a distinct square of illumination at the sample with an area of 1.1 μm^2^. To independently target nearby neurons in a moving worm, however, the neurons must be spatially separated by a sufficient distance. The minimum distance between targets depends on a variety of factors including the worms velocity, the system's latency and the ability of the system to infer the location of targeted neurons from the worm's outline, which can be susceptible to inhomogeneous compression and expansion of the worm body. We have shown the ability to illuminate two targeted neurons (ALM and AVM) spaced as close as 50 μm apart in a freely moving worm. Closer distances may be possible, however, especially in the dorsal-ventral dimension which is less susceptible to compression or expansion.

### 3.5. Fluorescence imaging

Red and green channel fluorescent images were recorded simultaneously on a Hamamatsu Orca Flash 4.0 siCMOS camera at 30 fps, with 33 ms exposure. To simultaneously image mCherry and GCaMP3 side-by-side we used a DV2 two-channel imager from Photometrics containing a custom filter set (Chroma #565dcxr dichroic, Semrock #FF01-609/54-25 red emission filter, Semrock #FF01-520/35-25 green emission filter). Fluorescent images were captured using HCImage software (Hamamatsu) running on a Dell Precision T7600 computer running Windows 7 with two Intel Xeon Quad Core 3.3 GHz processors and 49 GB of RAM.

### 3.6. Real-time computer vision software

An improved version of the MindControl software (Leifer et al., 2011), written in C, was used to generate patterned illumination and perform real-time feedback of the stage. The software was rewritten for 64-bit Windows 7. Additionally new features were added to give the user more options for performing timed stimulations and to give the user more control over stage feedback parameters. The overall stability of the software was also improved. The MindControl software ran on a second Dell Precision T7600 computer running Windows 7 with two Intel Xeon Quad Core 3.3 GHz processors and 16 GB of RAM. Source code is released under the GNU General Public License and is available for download on GitHub at https://github.com/leiferlab/mindcontrol.

### 3.7. Measuring latency

To measure the latency of the system, we we displayed a stationary image of a worm on an LCD computer monitor and imaged it with the system's behavior camera. A second high speed CMOS camera (Basler aca2000–300 km) and framegrabber (BitFlow Karbon) captured video with 1 ms exposure that simultaneously showed the LCD monitor, the DMD and a stopwatch (iPhone 5 s, Apple Computers). The MindControl software was run with the static worm as an input at the same settings as if we were conducting an optogenetic stimulus experiment. The latency was measured by counting the number of recorded frames between a stepwise translation of the static worm image on the monitor and the corresponding translation of the pattern of the DMD mirrors. The average framerate of the camera was calculated using the stopwatch.

### 3.8. Fluorescence imaging analysis

Neural activity is reported as normalized deviations from baseline of the ratio between GCaMP3 and mCherry fluorescence, $\frac{\Delta R}{R_{0}}=\frac{R-R_{0}}{R_{0}}$. The baseline *R*_0_ is defined as the mean of *R* during a time window at least 15 s in duration beginning with the start of the recording and ending with onset of optogenetic stimulation. We have chosen to report fluorescence intensity as a frame-by-frame ratio of green fluorescence from GCaMP3 to red fluorescence from mCherry so as to better account for artifacts from the animal's motion. The calcium insensitive mCherry serves as a built-in-control to compensate for slight changes in focus or from motion blur. The ratio $R=\frac{I_{\text{GCaMP3}}-B_{\text{green}}}{I_{\text{mCherry}}-B_{\text{red}}}$. The intensities *I*_GCaMP3_ and *I*_mCherry_ were measured as the median pixel intensity in the green and red channels, respectively, of the 40% brightest pixels of a circular region of interest (ROI) centered on the maximal intensity of a Gaussian smoothed image of the neuron pair AVA. The local background *B*_green_ and *B*_red_ in the green and red channels, respectively, were measured as the median pixel intensity in an annulus around the neuron. The ROI was selected in each frame by custom MATLAB scripts and confirmed by the user. The ROI for each neuron was a circle of radius 8 pixels and the background was an annulus of inner radius 20 pixels and an outer radius of 22 pixels. Images captured at 2048 × 2048 pixel resolution were binned to 1024 × 1024 pixels before analysis. The image scale was 0.62 μm/pixel during analysis. Custom MATLAB scripts were used to calculated the Δ*R*/*R*_0_ for each frame, and the timeseries was smoothed with a low-pass Gaussian filter (σ = 5 frames).

Transgenic animals expressing GFP instead of GCaMP3 were used to estimate the amount of observed calcium signals that could be attributed to motion artifact or instrument noise. GFP's fluorescence is insensitive to calcium activity, therefore signals of apparent calcium activity observed from GFP can be attributed to instrument noise or motion artifact. Animals expressing GFP were imaged under the same conditions as the GCaMP3 animals, however their reversals were spontaneous rather than induced. The apparent AVA activity of GFP controls is reported using the same Δ*R*/*R*_0_ metric except that GFP is used in place of GCaMP3.

### 3.9. Optogenetic-induced behavior experiments

Worms were grown on NGM agar plates seeded with 250 μL of OP50 *E. Coli* mixed with 1 μL of 1 mM all-trans retinal in ethanol solution. Plates were seeded on day 0, worms were transferred to seeded plates on day 1 and imaging was performed on day 2.

Spatially distinct regions of the worms body were defined to illuminate the cell bodies of selected neurons. The AVA region corresponded to 90% of the body width and 10% of the body length, centered 10% of the way from the anterior tip of the worm. The AVM region corresponded to 50% of the body width and 13% of the body length, centered 35.5% from the anterior tip, and 35% from the ventral edge. The ALM region was the same size, but centered 42.5% of the way from the anterior tip, and 35% from the lateral edge. Regions were selected to avoid overlap of processes, however the illumination region of AVA includes a small portion (estimated to be less then 20%) of the ALM process.

To mitigate suspected worm-to-worm variability in ChR2 expression and retinal uptake, we selected worms for our experiment that reversed in response to whole head illumination but did not reverse or pause in response to illuminating the small portion of the ALM process near AVA. Previously we had observed that illuminating small areas of neuronal processes alone usually caused little effect (Leifer et al., 2011). Approximately three quarters of worms tested responded with reversals to a brief whole head illumination, and of those, 39 of 53 worms had no noticeable response to the onset of process illumination and were thus deemed suitable for experimentation. Whole head illumination was performed manually on a fluorescent dissection scope (Nikon SMZ-1500) while process illumination was performed on the main instrument by illuminating the AVA region.

For imaging, worms were washed in M9 solution, transferred to approximately 1.5-mm thick NGM agarose plates and covered with mineral oil to improve contrast under dark-field illumination. Imaging began after allowing animals to acclimate for 5 min. Optogenetic stimulation was delivered after >15 s of AVA imaging. Each stimulus constitutes a separate trial. For most experiments animals were stimulated once and immediately discarded. Some worms were stimulated twice with a 3 min rest in between. No worm was stimulated more than twice. Trials where the worm underwent spontaneous reversals prior to stimuli, or prolonged bouts of multiple distinct reversals in response to the stimuli were excluded from analysis.

Occasionally during an experiment the real-time computer vision software transiently fails to correctly segment the worm's head and tail. This often occurs when the worm touches itself during the deepest ventral bend portion of an omega turn or when the worm encounters a bubble or other visual artifact in the agarose plate. These periods of segmentation failure are shown as gaps in the behavioral trace, as visible in Supplementary Figure 1.

Behavioral data and calcium activity data were analyzed using custom MATLAB scripts, available at http:://github.com/leiferlab/dualmag-analysis. Each trial was manually classified as reverser or non-reverser. Trials were classified as non-reversers if the animals's velocity failed to drop below zero in a short time window after stimulus.

An empirical model of AVA activity was fit to each calcium trace to extract a characteristic amplitude of calcium activity and to define a time region over which to average the velocity (Supplementary Figure 1). This method was chosen because it allows us to compare velocities and calcium amplitudes in a uniform way across trials even when the response varies from trace to trace or even when the worm fails to reverse. Calcium levels in AVA were assumed to increase and then decrease in a similar manner to the voltage across a capacitor in an *RC* circuit in response to a voltage square wave,

(1) $$f(x)=\{\begin{array}{ll} 0 & \text{if }0\leq t\\ A(1-e^{-4t/\tau_{1}}) & \text{if }0<t\leq \tau_{1}\\ Ae^{-(t-\tau_{1})/(\tau_{2}-\tau_{1})} & \text{if }\tau_{1}<t \end{array}\text{​}$$

where the onset of optogenetic stimulus occurs at *t* = 0, *A* is the amplitude of neural activity and τ_1_ and τ_2_ are the timescales for calcium increase and decrease and are allowed to vary between trials. Mean velocity was calculated over the time window from stimulus onset (*t* = 0) to τ_1_. The parameters *A*, τ_1_ and τ_2_ were fit for each trace using least-mean squared and the Nelder-Mead method.

## 4. Discussion

The optical neurophysiology system presented here combines targeted optogenetic stimulation with calcium imaging in a freely behaving unrestrained animal. We used the system to investigate how mechanosensory signals from two neuronal cell types in *C. elegans* are integrated into a downstream command interneuron and how their combined activity influences behavior. The experiments performed here could not be done using any previously available method.

A crucial feature of the current system is its ability to independently deliver light to targeted neurons expressing either ChR2 or GCaMP3. The current system, however, cannot independently image a neuron that co-expresses both GCaMP3 and ChR2 together because the two protein's excitation spectra overlap. Nonetheless, with minor modifications, the current system could be adapted to do so by using recently developed red-shifted ChR2 or RCaMP variants (Zhang et al., 2008; Erbguth et al., 2012; Akerboom et al., 2013) such that their excitation spectra no-longer overlap.

A consensus has emerged that there is a distinct need for the development of new methods to manipulate and monitor neural activity in behaving animals at cellular resolution and circuit scale (NIH, 2013). The work here represents an important step toward that goal and is the first instance, in any organism, of non-invasive simultaneous optical manipulation and calcium imaging in an unrestrained animal.

## Author contributions

Andrew M. Leifer designed the instrument. Andrew M. Leifer and Frederick B. Shipley built the instrument. Christopher M. Clark and Frederick B. Shipley generated transgenic animals. Andrew M. Leifer, Frederick B. Shipley, Christopher M. Clark, and Mark J. Alkema designed experiments. Frederick B. Shipley conducted all experiments.

## Conflict of interest statement

The authors declare that the research was conducted in the absence of any commercial or financial relationships that could be construed as a potential conflict of interest.

## Supplementary material

Supplementary Movie 1 **(Smovie1.mp4) Movie, MPEG-4 (10 MB).** A control worm expresses GFP in interneuron type AVA and in the mechanosensory neurons including ALM and AVM (*(zfEx416)* [P*rig3::GFP::SL2::mCherry*], *(zdIs5)* [P*mec- 4::GFP*]). Different combinations of ALM, AVM, and AVA + pharyngeal neurons are illuminated independently. Arrow indicates location of AVA by nearby pharyngeal neurons I1, I4, M4, and NSM. The text indicates targeted neuron. No fluorescence is observed in non-targeted regions.

Supplementary Movie 2 **(Smovie2.mp4) Movie, MPEG-4 (20 MB).** Calcium dynamics of command interneuron type AVA are shown before, during and after a 2.7 s stimulation to mechanosensory neuron ALM. The transgenic animal shown here expresses GCaMP3 and mCherry in interneuron AVA and ChR2 in the soft-touch mechanosensory neurons *(zfIs18)* [P*mec-4::ChR2::YFP*]; (zfIs42) [P*rig-3::GCaMP3::SL2::mCherry*]. Upper left panel shows green fluorescence from GCaMP3 in false color. The upper middle panel shows red fluorescence from mCherry in false color. Orange circle indicates location of command interneuron AVA. The upper right panel shows worm behavior. Green circle indicates head. Red triangle indicates tail. The animals outline is shown in yellow. Blue area shows region of blue laser light illumination. Lower panel shows calcium activity. Stimulus onset occurs at *t* = 0 s. The trial shown here is the same as that in Figure 2B. Background fluorescence that is visible in the red channel is presumably due to non-specific expresion of mCherry. It is thought that the Sl2 sequence that precedes mCherry acts as a weak non-specific promotor in addition to its desired function as a post-translational splice site. This background fluorescence is accounted for when calculating Δ*R*/*R*_0_.

Supplementary Figure 1 **Calcium dynamics of command interneuron AVA (green) and animal velocity (gray) are shown before, during and after a 2.7 s stimulation to mechanosensory neurons ALM and AVM for each trial.** All trials that resulted in a reversal are shown (9 animals, one trial per animal). The transgenic animals shown here express GCaMP3 and mCherry in interneuron AVA and ChR2 in the soft-touch mechanosensory neurons *(lin-15(n765ts))*; (zfIs18) [P*mec-4::ChR2::YFP*]; (zfis42) [P*rig-3::GCaMP3::SL2::mCherry*]. Stimulus onset occurs at *t* = 0 s (first dashed vertical line) and extends for 2.7 s (blue shaded region). An empirical three-parameter model of AVA activity is fit to each calcium transient (blue dashed curve). The fit parameter τ_1_ is shown as vertical dashed black line. The average velocity for each trace, as shown in Figures 3B, 4A, is calculated by averaging the velocity between *t* = 0 and *t* = τ_1_ (region between vertical black dashed lines.)

Supplementary Figure 2 **GFP control.** Green fluorescence from animals expressing GFP instead of GCaMP3 in AVA was observed (green line) during spontaneous reversals (QW1075 *(zfEx416)* [P*rig-3::GFP:SL2::mCherry*], *(zdIs5)* [P*mec-4::GFP*]). An example trace is shown (*n* = 5 trials). The y axis has been scaled for comparison to Figure 2B. Δ*R*/*R*_0_ is calculated as before, but with GFP fluorescence instead of GCaMP3. Because GFP is insensitive to modest changes in calcium levels, fluctuations in the signal observed here is presumably due to motion artifact or instrument noise.
