# Supplementary figures and images for: Simultaneous optogenetic manipulation and calcium imaging in freely moving C. elegans
Source: Front Neural Circuits. 2014 Mar 24;8:28. doi: 10.3389/fncir.2014.00028 (PMC3970007; doi:10.3389/fncir.2014.00028)

Supplementary Figure 1

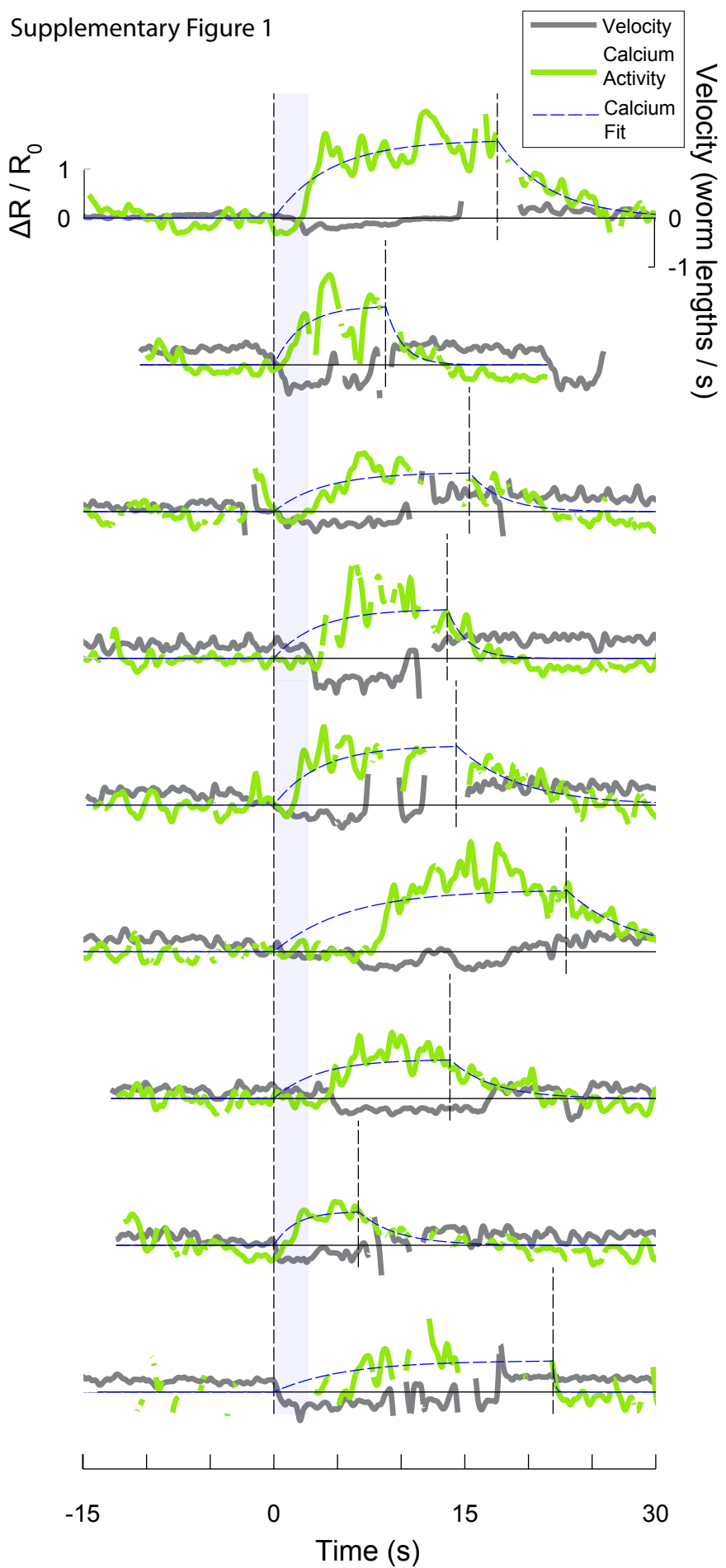

Supplement: Supplementary Movie 1 — (Smovie1.mp4) Movie, MPEG-4 (10 MB). A control worm expresses GFP in interneuron type AVA and in the mechanosensory neurons including ALM and AVM ((zfEx416) [Prig3::GFP::SL2::mCherry], (zdIs5) [Pmec- 4::GFP]). Different combinations of ALM, AVM, and AVA + pharyngeal neurons are illuminated independently. Arrow indicates location of AVA by nearby pharyngeal neurons I1, I4, M4, and NSM. The text indicates targeted neuron. No fluorescence is observed in non-targeted regions. [file Presentation1.ZIP › Leifer-Presentation 1/Leifer_Supplementary Figure_1.PDF]

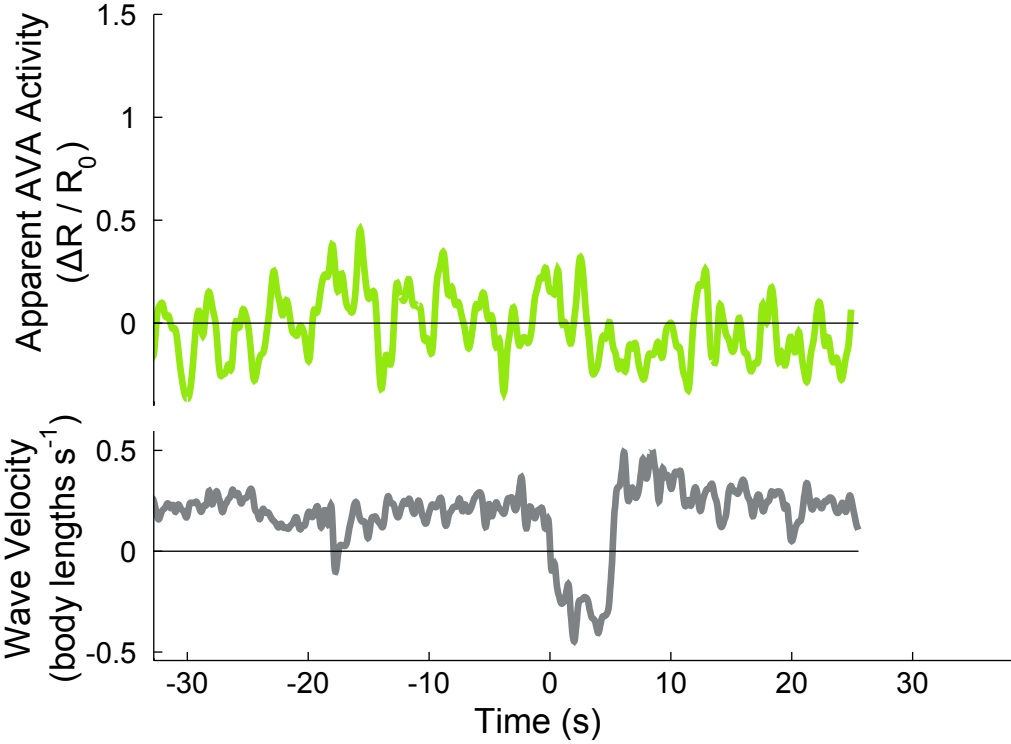

Supplement: Supplementary Movie 1 — (Smovie1.mp4) Movie, MPEG-4 (10 MB). A control worm expresses GFP in interneuron type AVA and in the mechanosensory neurons including ALM and AVM ((zfEx416) [Prig3::GFP::SL2::mCherry], (zdIs5) [Pmec- 4::GFP]). Different combinations of ALM, AVM, and AVA + pharyngeal neurons are illuminated independently. Arrow indicates location of AVA by nearby pharyngeal neurons I1, I4, M4, and NSM. The text indicates targeted neuron. No fluorescence is observed in non-targeted regions. [file Presentation1.ZIP › Leifer-Presentation 1/Leifer_Supplementary Figure_2.PDF]
